# High‐concentration hydrogen inhalation mitigates sepsis‐associated encephalopathy in mice by improving mitochondrial dynamics

**DOI:** 10.1111/cns.70021

**Published:** 2024-09-11

**Authors:** Yan Cui, Shuqi Meng, Nannan Zhang, Jingya Liu, Lina Zheng, Wanjie Ma, Yu Song, Zhiwei Wang, Yuehao Shen, Jianfeng Liu, Keliang Xie

**Affiliations:** ^1^ Department of Pathogen Biology School of Basic Medical Sciences, Tianjin Medical University Tianjin China; ^2^ Department of Critical Care Medicine Tianjin Medical University General Hospital Tianjin China; ^3^ Department of Anesthesiology Tianjin Institute of Anesthesiology, Tianjin Medical University General Hospital Tianjin China

**Keywords:** hydrogen, mitochondrial biogenesis, mitochondrial dynamics, sepsis‐associated encephalopathy

## Abstract

**Background:**

Sepsis‐associated encephalopathy (SAE) is a neuronal injury with poor prognosis. Mitochondrial dysfunction is critical in SAE development, and hydrogen gas (H_2_) has a protective effect on septic mice. This study aimed to investigate the effect of high concentration (67%) of H_2_ on SAE and whether it is related to mitochondrial biogenesis and mitochondrial dynamics.

**Methods:**

A mouse sepsis model was induced by cecal ligation and puncture. The mice inhalated 67% H_2_ for 1 h at 1 and 6 h post‐surgery, respectively. The 7‐day survival rate was recorded. Cognitive function was assessed using the Y‐maze test and Morris water maze test. Serum inflammatory factors, antioxidant enzymes, as well as mitochondrial function indexes including mitochondrial membrane potential (MMP) and ATP in the hippocampal tissue were evaluated 24 h after surgery. Mitochondrial dynamic proteins (DRP1 and MFN2) and biosynthetic proteins (PGC‐1α, NRF2, and TFAM) in the hippocampal tissue were detected. Moreover, the morphology of mitochondria was observed by transmission electron microscopy.

**Results:**

Inhalation of 67% H_2_ improved the 7‐day survival rates and recognition memory function of septic mice, alleviated brain antioxidant enzyme activity (SOD and CAT), and reduced serum proinflammatory cytokine levels. H_2_ inhalation also enhanced the expression of MFN2 and mitochondrial biogenesis‐related factors (PGC‐1α, NRF2, and TFAM) and decreased the expression of fission protein (DRP1), leading to improvement in mitochondrial function, as evidenced by MMP and ATP levels.

**Conclusions:**

Inhalation of high concentration (67%) of H_2_ in septic mice improved the survival rate and reduced neuronal injury. Its mechanism might be mediated by enhancing mitochondrial biogenesis and mitochondrial dynamics.

## Graphical abstract

High concentration (67%) of hydrogen gas inhalation exerts a protective effect on sepsis‐associated encephalopathy, and the mechanism may be improving mitochondrial function through the promotion of mitochondrial biogenesis and the improvment of mitochondrial dynamics.

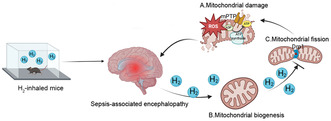

## 1 INTRODUCTION

Sepsis is a severe condition marked by inadequate host response to infection, causing multi‐organ dysfunction.^1^ The latest Sepsis 3.0 guidelines underscore the importance of studying organ dysfunction in sepsis. Brain damage is a prevalent organ dysfunction associated with sepsis, which leads to neurological complications such as intensive care unit‐acquired paralysis^2^ and cognitive impairment,^3^, ^4^ which impairs functional status and quality of life.

Mitochondria are dynamic and multifunctional organelles that are crucial for eukaryotic cells as they facilitate biological oxidation and energy conversion through oxidative phosphorylation.^5^ This process generates ATP, which is essential for cellular metabolism, physiology, and organismal functions. Moreover, mitochondria regulate intracellular calcium levels, generate reactive oxygen species (ROS), and modulate cellular signaling and redox processes. Changes in the structure of mitochondria affect energy production, so the dynamics such as fusion, division, motility, and positional binding need to be controlled.^6^ Despite mitochondrial protein depletion, sepsis survivors with multiple organ dysfunction exhibit heightened mitochondrial biosynthesis and antioxidant defense responses, preserving functional and energetic status.^7^ However, continuous mitochondrial damage and ensuing inflammation can cause severe outcomes such as cell death, multiple organ failure, and long‐term cognitive dysfunction,^8^, ^9^, ^10^ which are associated with sepsis prognosis and encephalopathy.^11^ Hydrogen gas (H_2_), known for its properties such as scavenging ROS and modulating immune responses, has been studied as a therapeutic molecule.^12^ Our research team has demonstrated the efficacy of low concentration (2%) of H_2_ in treating sepsis and multiorgan failure using animal and cellular models.^13^ Recently, high concentration of H_2_ therapy devices has shown promising results in treating various diseases.^6^, ^14^ However, the therapeutic effect of high‐concentration of H_2_ on SAE is still unclear.

Therefore, this study aimed to evaluate the effectiveness of inhaled high‐concentration of H_2_ in mitigating SAE and whether the mechanism was related to mitochondrial dynamics and biogenesis.

## 2 MATERIALS AND METHODS

### 2.1 Animals

Male C57BL/6J mice, weighing 20–25 g and aged 6–8 weeks, were acquired from the Experimental Animal Centre of the Chinese Academy of Military Medical Sciences, Beijing, China. After an acclimation period, the mice were housed in a pathogen‐free environment with ad libitum access to food and water (temperature of 20–22°C, humidity of 30%–70%, and 12‐h cycle of light and darkness). Ethical regulations were followed throughout all experimental procedures, which received the approval of the Experimental Animal Management Committee of Tianjin Medical University (Grant No. IRB2021‐DWFL‐274).

### 2.2 Cecal ligation and puncture (CLP)

Sepsis model was established by cecal ligation and puncture. After anesthesia with 2% isoflurane, a 1 cm incision was made in the middle of the abdomen. The cecum was ligated with surgical sutures at the end of one‐half of the distance below the ileocecal flap, and a sterile 20 G needle was employed to perforate the midpoint of the ligated cecum and extrude a small amount of bowel contents. After returning both the cecum and extruded contents to the abdominal cavity, the abdominal wall incision was closed using sterile 6‐0 silk in layers. Subsequently, a post‐surgery subcutaneous injection of 5 mL/100 g saline was administered.

### 2.3 Experimental procedures

**FIGURE 1:**
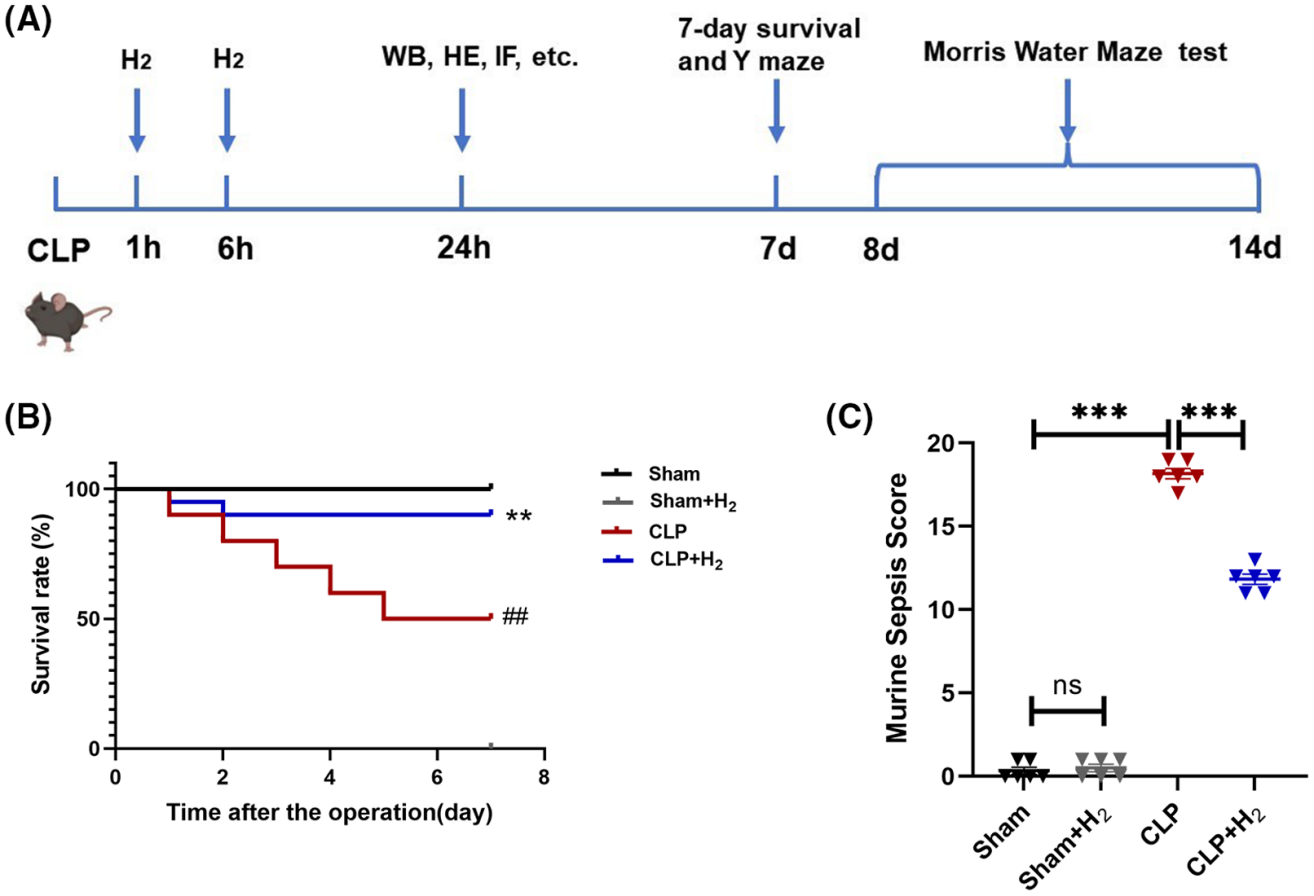
67% H_2_ inhalation improved the 7‐day survival rate and MSS score of septic mice. (A) Experimental flow chart. (B) 7‐day survival rate of mice; the values are expressed as the survival rates (*n* = 20, log‐rank test). ^##^ *p* < 0.01 vs. Sham group, **p < 0.01 vs. CLP group. (C) Murine sepsis score (*n* = 6). ***p < 0.001 compared between two groups.

The mice were randomly assigned into four groups (*n* = 62 per group): Sham group (Sham), Sham with 67% hydrogen group (Sham+H_2_), CLP group (CLP), and CLP with 67% hydrogen group (CLP + H_2_). The Sham group and Sham + H_2_ group only laparotomy without ligation puncture cecum. The sepsis model was induced using the CLP procedure in the CLP group and the CLP + H_2_ group. The Sham+H_2_ group and CLP + H_2_ group inhaled high concentration of H_2_ for 1 h at 1 and 6 h after the operation while the remaining groups only inhaled air. The survival rates of the mice (*n* = 20 per group) were monitored for 7 days post‐operation. After 24 h of molding, six mice from each group were randomly selected for subsequent hippocampal damage assessment and their serum was used for the detection of serum inflammatory factors. In addition, 30 mice in each group were selected to take their hippocampus for detection of antioxidant enzyme activity (*n* = 6), mitochondrial membrane potential (MMP) (*n* = 6), ATP content (*n* = 6), Mitochondrial morphology (*n* = 6) and Western blotting (*n* = 6). In addition, the Y‐maze test (*n* = 6) and the Morris water maze test (*n* = 6) were performed on the 7th and 8th postoperative days, respectively (Figure 1A).

### 2.4 Hydrogen gas treatment

The mice in the Sham+H_2_ group and CLP + H_2_ group were exposed to 67% hydrogen gas and 33% oxygen mixture using an AMS‐H‐01 hydrogen gas and oxygen atomizer (Shanghai Asclepius Meditec Co., Shanghai, China) and placed in the sealed box. A layer of calcium lime was placed at the bottom of the box to absorb exhaled CO_2_. Prior to the experiment, the box was charged with the hydrogen‐oxygen mixture for 30 min. Continuous monitoring of H_2_ concentration was performed using a heat tracing gas analyzer (Thermal Fisher, Waltham, MA, USA). In the Sham+H_2_ and CLP + H_2_ groups, the hydrogen inhalation was exposed to the appropriate concentration for 1 h at 1 and 6 h post‐operation, respectively. The Sham group and CLP group received only air.

### 2.5 Y Maze test

Y‐maze recognition memory experiments were conducted at 7th day after surgery to evaluate cognitive function. The Y‐maze, provided by Shanghai Xinsoft Information Technology Co., consisted of three arms (A, B, and C) positioned at a 120° angle to each other. During the experiments, mice underwent a 10‐min acclimation period, after which arm C was obstructed with a baffle. Mice were then free to explore arms A and B while various parameters such as distance, time, speed, entry count, latency, and resting time were tracked using a video recording system. The maze was disinfected using 75% ethanol to eliminate residual odors before removing the baffle from arm C after 1 h of training. Mice were subsequently placed at arm A and allowed to move freely for an additional five minutes, monitored by the video acquisition system. Incidences of mouse entry into each zone served as indicators of mobility.

### 2.6 Morris water maze (MWM) test

The MWM experiment was carried out in a round plastic tank (90 cm in diameter and 50 cm in height) containing titanium dioxide at a water temperature of 23°C. Titanium dioxide made water opaque and was used to track mice. The pool is divided into four quadrants, one of which places a circular platform 2 cm below the surface of the water. In the first 6 days of training, the mouse was randomly placed in the quadrant of the pool to allow the mice to freely find the platform. When the mouse could not find the platform within 60 s, it was led to the platform to rest for 10 s. On the seventh day, the platform was removed from the pool and the mice were placed in the water to swim freely for 60 s. The number of platform crossings and the residence time in the quadrant where the platform was located were recorded.

### 2.7 Brain histopathological detection

After 24 hours of molding, six mice from each group were randomly selected for subsequent hippocampal damage assessment. The brain tissue was fixed in 4% paraformaldehyde at 4°C overnight. One part of the brain tissue was used for Nissl staining and the other part was used for HE staining. Following transcardial perfusion with 4% paraformaldehyde, the tissues were fixed in 10% formalin for 24 h and embedded in paraffin. After the 10 μm slices were dewaxed, rehydrated with xylene and ethanol, they were stained with Nissl and HE, respectively. The neuronal organization in the CA1 hippocampal region and niche morphology were scrutinized under a light microscope (Olympus, Japan). Three sections were randomly selected from each sample, and three fields of view were observed per section by two pathologists who did not know the experiment. The normal spinal neuron count was quantified and averaged using Image pro‐plus 6.0 image analysis software.

### 2.8 Immunofluorescence

Mouse brain was placed in 4% paraformaldehyde at 4°C overnight. After dehydration, OCT reagent (4593, Solarbio, China) was used for embedding. Then, the embedding block was cut into 10 μm slices. The primary antibody Drp1 (1:300, sc‐271583, SantaCruz, USA) and Nrf2 (1:300, 80593‐1‐RR, Proteintech, China) were added to the slice separately at 4°C overnight. On the next day, fluorescent secondary antibody was added to the sections. Then the sections were observed under the fluorescence microscope (Olympus, Japan).

### 2.9 Serum pro‐inflammatory factors

At the 24‐h post‐surgery, approximately 0.6 mL blood samples were collected via the orbital vein from a random selection of six mice in each group. The samples were centrifugated at 4000 ***g*** for 10 minutes to obtain serum. ELISA analysis was used to measure serum concentrations of TNF‐α (MTA00B, R&D Systems, USA), IL‐1β (MLB00C, R&D Systems, USA), and HMGB1 (SP14752, Saipei, China).

### 2.10 Detection of antioxidant enzyme activity

At the 24‐hour after operation, six mice from each group were randomly selected, and their hippocampal tissue was extracted. The activity of Superoxide Dismutase (SOD) and Catalase (CAT) was evaluated using a 722 visible spectrophotometer (Shanghai Analytical Instrument Factory) with Cayman kits obtained from the USA.

### 2.11 JC‐1 assay of the mitochondrial membrane potential (MMP)

At the 24‐h after operation, the hippocampus of six mice in each group was taken and mixed with mitochondrial isolation medium A for homogenization. The homogenate underwent centrifugation at 1000 ***g*** for 10 min at 4°C, and the supernatant was discarded. The collected mitochondria were obtained from the precipitate after centrifugation at 11,000 ***g*** for 10 min at 4°C. Protein concentration was determined by adding preservation medium (400 μL per gram of tissue), and MMP was measured using red and green fluorescence intensity. JC‐1 staining solution (0.9 mL) was mixed with the mitochondria, and excitation wavelengths of 490 and 525 nm were measured using an EnSpire multifunctional enzyme marker (PerkinElmer, USA). Emission wavelengths of 530 and 590 nm were utilized, respectively.

### 2.12 Measuring ATP content

After 24‐h post‐surgery, hippocampal tissue was extracted from six randomly selected mice in each group. The tissue was added to the ATP detection lysate (Biyuntian Institute of Biotechnology) and homogenized 10 times with a precooled glass homogenizer. The resulting homogenate was centrifuged at 12,000 ***g*** for 5 min at 4°C, and the supernatant was discarded. Subsequently, 20 μL of tissue samples were mixed with an EnSpire multifunctional enzyme marker (PerkinElmer, USA) to determine relative para‐optical unit values in the assay wells. Finally, the ATP content was calculated using the resultant standard curve.

### 2.13 Western blotting

Hippocampal tissues were retrieved from an ultralow temperature refrigerator, weighed, and placed in precooled PBS buffer with protease inhibitor. After a 5‐min homogenization using an ultrasonic cell crusher, the samples were centrifuged at 15,000 ***g*** for 10 min at 4°C. The resulting supernatant underwent BCA protein quantification, followed by the addition of protein loading buffer, boiling, denaturation, and storage at −80°C. Following electrophoretic separation and membrane transfer, the membranes were blocked with skimmed milk and incubated overnight at 4°C with primary antibodies against Drp1 (1:1000, Abcam, USA), Mfn2 (1:1000, Abcam, USA), PGC‐1α (1:1000, Abcam, USA), NRF2 (1:1000, Abcam, USA), TFAM (1:1000, Abcam, USA), and GAPDH (1:10,000, Abcam, USA). Subsequently, the membranes underwent five minutes washes with TBST, followed by incubation with goat anti‐rabbit secondary antibody (1:5000, Affinity, Australia) at room temperature for one hour. Expression levels were determined by analyzing the gray values using Image J software, and the ratio of the target protein's gray value to that of the reference GAPDH band was used as a reflection of expression level.

### 2.14 Transmission electron microscope observation

The hippocampal tissue was cut into small pieces and put them in 2.5% glutaraldehyde at 4°C overnight. After fixation in 1% OsO4 and alcohol dehydration, tissues were embedded with Epon‐Araldite resin. Then, the embedding block was cut into 50 μm ultrathin sections and stained sequentially with 2% uranyl acetate and lead solution. Finally, the sections were observed with the transmission electron microscope (Hitachi, Ltd., Tokyo, Japan).

### 2.15 Statistical analysis

The statistical analysis was performed using *SPSS* 19.0 software. Shapiro–Wilk test was used to test the positive pacificity of the data. Normally distributed data were presented as mean ± standard deviation ($\bar{x}\pm s$), and survival rates were expressed as percentages. One‐way anova was used to analyze measurement data, while the survival rate was analyzed by Kaplan–Meier method and log‐rank test. Statistical significance was determined at *p* < 0.05.

## 3 RESULTS

### 3.1 High concentration of H_2_ inhalation improved 7‐day survival rate and MSS score of sepsis mice

As shown in Figure 1B, the Sham and Sham + H_2_ groups maintained 100% survival over the 7‐day period. Compared with the Sham group, septic mice exhibited a 50% 7‐day survival rate (*p* < 0.01), which significantly improved to 90% after 67% H_2_ inhalation (*p* < 0.01). Inhalation of 67% H_2_ notably enhanced the 7‐day survival rate of septic mice. In addition, MSS scores were also performed on sepsis mice (Figure 1C). There was no significant difference between the Sham and the Sham+H_2_ groups (*p* > 0.05), while the MSS score of the CLP group was significantly increased (*p* < 0.001), and the MSS score decreased in the CLP + H_2_ group (*p* < 0.001).

### 3.2 High concentration of H_2_ inhalation ameliorated cognitive dysfunction

**FIGURE 2:**
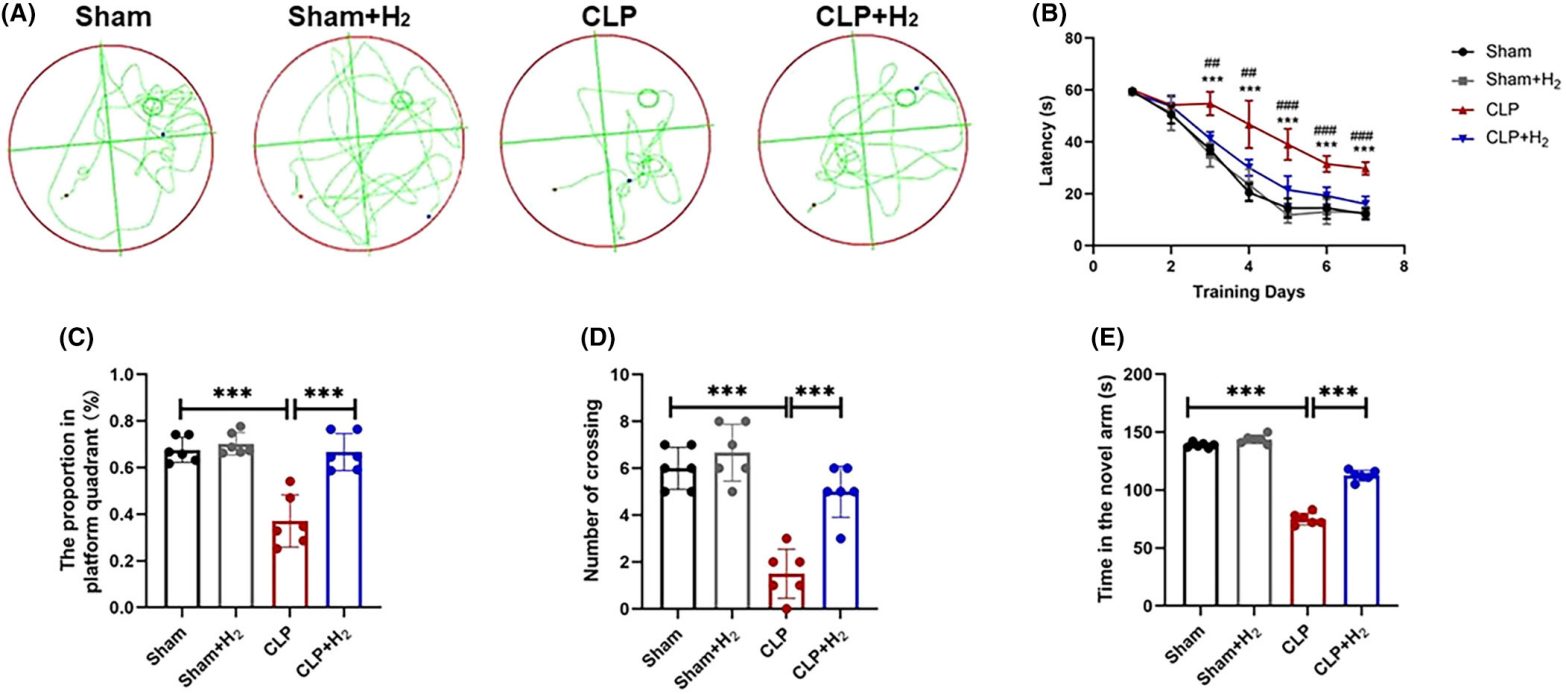
67% H_2_ inhalation improved cognitive dysfunction in mice with sepsis. (A) Trajectory plot of the seventh day after training. (B) Escape latency during training in mice of each group. ***p < 0.01 vs. Sham group, ^##^ *p* < 0.01, ^###^ *p* < 0.001 vs.CLP group. (C) Time ratio of mice in the quadrant where the platform is located on 7th day after training. (D) The number of times the mice crossed the platform on 7th day after training. (E) Times in the novel arms in the Y‐maze test. All the data were expressed as means ± SD (*n* = 6 per group). ***p < 0.001 compared with two groups.

Recognition memory of the mice was assessed using morris water maze (MWM) test and Y‐maze test. As the number of training increases, the time for mice to find a platform gradually decreased. Notably, mice in the CLP group took longer to find a platform than in the Sham group during the same training time (*p* < 0.001). However, the time to find the platform was reduced after H_2_ therapy (*p* < 0.01). After removing the platform on the seventh day, the proportion of mice staying on the platform (Figure 2C) and the number of times they crossed the platform (Figure 2D) were reduced compared with the Sham group (*p* < 0.001), while the CLP + H_2_ group significantly increased both (*p* < 0.001). In addition, the Y maze results showed that the CLP group demonstrated significantly reduced C‐arm dwell times compared to the Sham group (*p* < 0.001), while the Sham + H_2_ group showed no significant differences compared to the Sham group (*p* > 0.05). The CLP + H_2_ group exhibited a significant increase in C‐arm dwell times relative to the CLP group (*p* < 0.001) (Figure 2E).

### 3.3 High concentration of H_2_ inhalation significantly reduced brain injury

**FIGURE 3:**
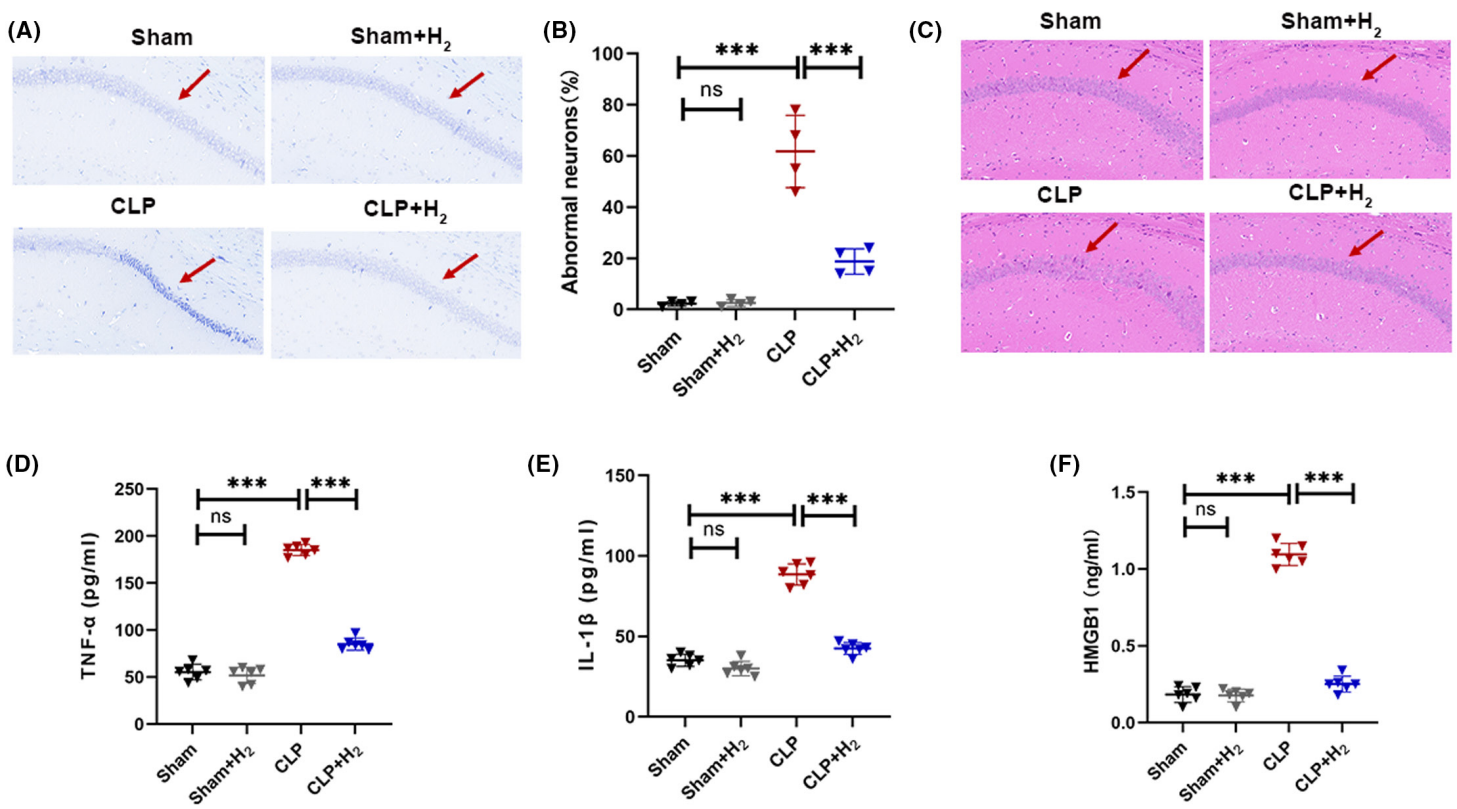
67% H_2_ inhalation ameliorated brain injury in septic mice. (A) Nissl staining of hippocampus (×200). (B) The number of abnormal neurons was calculated based on Nissl staining. (C) HE staining of brain tissue. (D–F) Serum TNF‐α, IL‐1β, and HMGB1. All the data were expressed as means ± SD (*n* = 6 per group). The red arrows represent the obvious differences. ***p < 0.01 compared with two groups.

As Shown in Figure 3A, the CA1 area of the hippocampus in the Sham and Sham + H_2_ groups displayed well‐organized neuronal cells with neat arrangements and numerous, large, purple‐blue Nissier vesicles, while the CLP group showed sparse neuronal cell structure with significantly reduced normal neurons, irregular arrangements, and reduced Nissier vesicle counts. Moreover, mice in the CLP + H_2_ group exhibited slightly improved neuronal arrangements with reduced pathological changes and significantly lower apoptotic neuron counts compared to the CLP group (*p* < 0.001) (Figure 3B). In HE staining (Figure 3C), the hippocampal pyramidal neurons in the Sham group and the Sham+H_2_ group were well‐structured and tightly arranged. In the CLP group, the nuclear nucleus of hippocampal pyramidal neurons was condensed, the cytoplasm was hyperchromatic, and the arrangement was disordered. After treatment with H_2_, the morphology and arrangement of hippocampal pyramidal neurons improved. For the detection of inflammatory factors (Figure 3D–F), TNF‐α, (*p* < 0.001), IL‐1β (*p* < 0.001) and HMGB1 (*p* < 0.001) were significantly increased in the CLP group compared with the Sham group, while these inflammatory factors were significantly reduced in the CLP + H_2_ group (*p* < 0.001).

### 3.4 High concentration of H_2_ alleviated mitochondrial dysfunction

**FIGURE 4:**
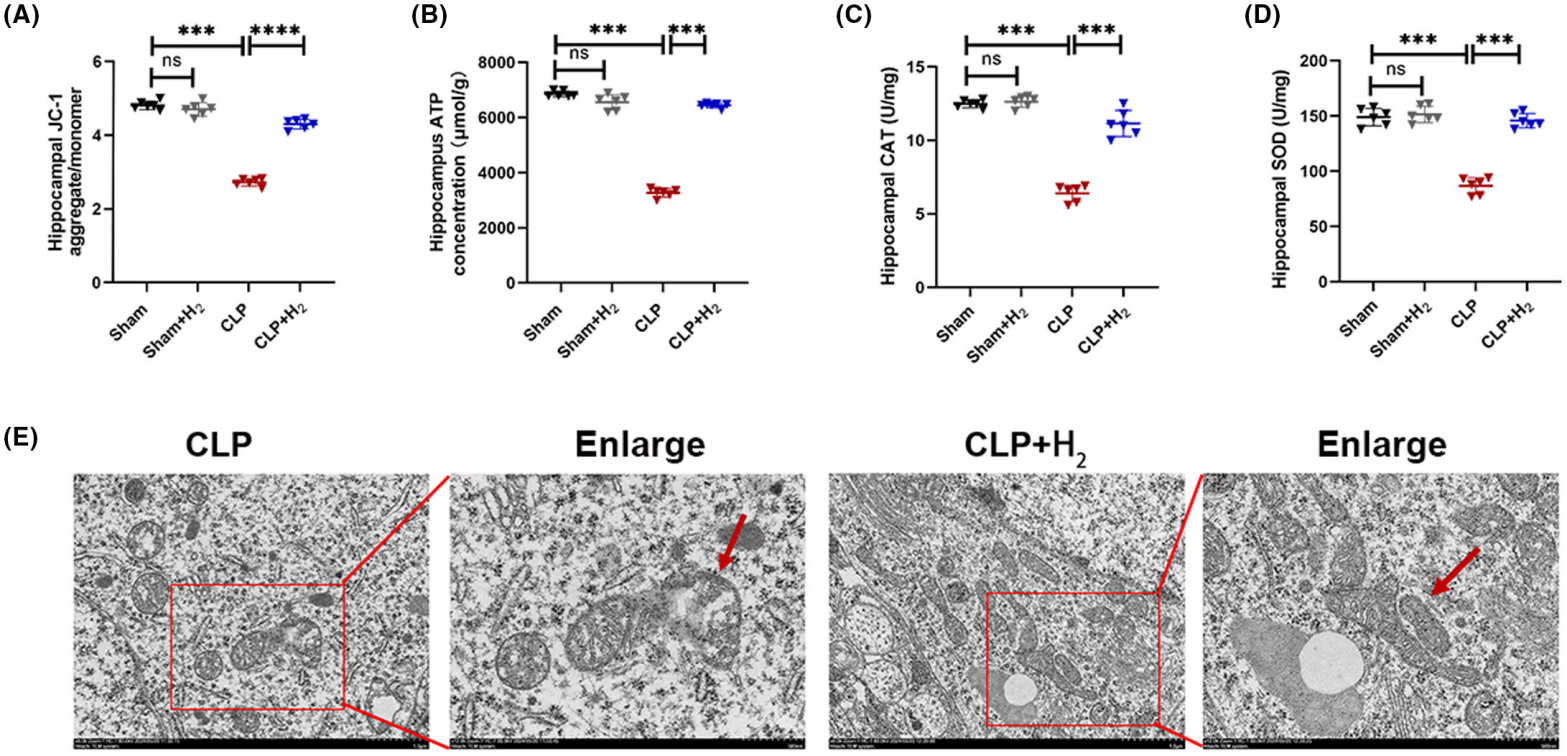
67% H_2_ inhalation alleviated mitochondrial dysfunction of septic mice. (A) The JC‐1 aggregate/monomer ratio was used to assess the MMP. (B) The ATP content was detected based on fluorescein. (C) Hippocampal CAT. (D) Hippocampal CAT. (E) The morphology of mitochondria was observed by transmission electron microscopy. The red arrows represent the obvious differences. All the data were expressed as means ± SD (*n* = 6 per group). ***p < 0.01 compared with two groups.

MMP is essential for ATP generation. In the present study (Figure 4A,B), MMP and ATP levels were significantly reduced in the CLP group compared to the Sham group (*p* < 0.001), indicating that mitochondrial function was impaired due to sepsis. However, the CLP + H_2_ group exhibited a significant increase in MMP and ATP compared to the CLP group (*p* < 0.001). At the same time, the activities of antioxidant enzymes SOD and CAT in the hippocampus were also detected (Figure 4C,D). We observed that a significant decrease in SOD and CAT activity (*p* < 0.001) in the hippocampus tissue than those of the Sham group, which was reversed by inhalation of 67% H_2_ (*p* < 0.001). Moreover, transmission electron microscopy was used to observe the morphology of mitochondria directly. As shown in Figure 4E, Mitochondrial morphology was abnormal in the CLP group, including mitochondrial swelling, ridge collapse and mitochondrial membrane rupture. After H_2_ therapy, mitochondrial damage was significantly reduced.

### 3.5 High concentration of H_2_ improved mitochondrial dynamics

**FIGURE 5:**
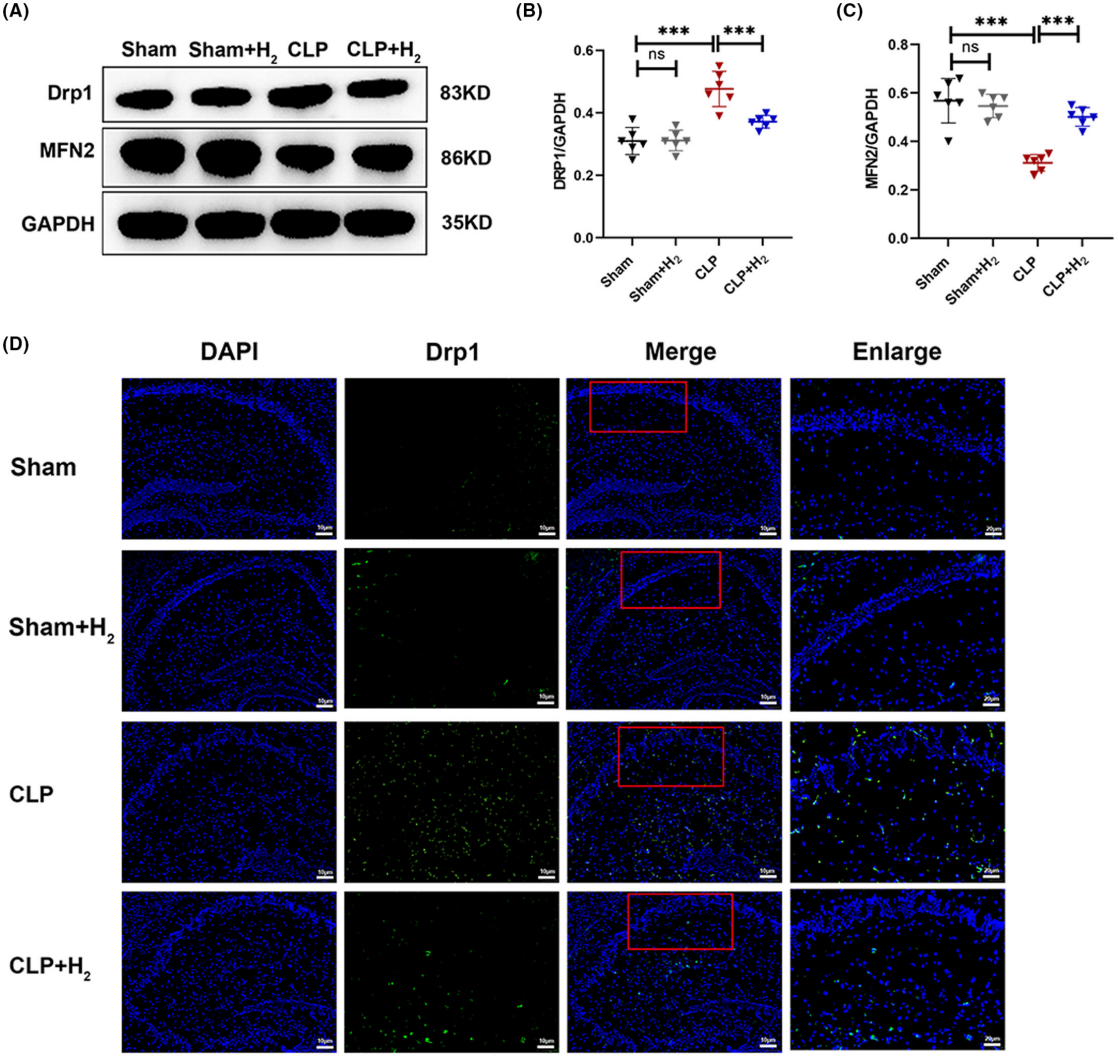
67% H_2_ improved the mitochondrial dynamics in septic mice. (A–C) The expression of DRP1 and MFN2 was detected by western blotting. (D) Drp1 was observed by immunofluorescence staining. All the data were expressed as means ± SD (*n* = 6 per group). ***p < 0.01 compared with two groups.

GTPases play a predominant role in regulating mitochondrial fusion, while Drp1 and Mfn1/Mfn2 proteins are essential for both mitochondrial fission and fusion. In the CLP group, there was a significant increase in DRP1 expression (*p* < 0.001), whereas Mfn2 expression showed a significant decrease (*p* < 0.001). In contrast, the CLP + H_2_ group displayed a significant decrease in DRP1 protein expression (*p* < 0.001) and a notable increase in MFN2 protein expression (*p* < 0.001) compared to the CLP group (Figure 5A–C). In addition, Drp1 was labeled with green fluorescence and directly observed by immunofluorescence (Figure 5D). Compared with the Sham group, the expression of Drp1 in the CLP group was significantly increased, while, the expression of Drp1 decreased significantly after H_2_ treatment. Therefore, CLP led to a substantial imbalance between mitochondrial fusion and fission in hippocampal tissue, which was improved by exposure to high levels of H_2_.

### 3.6 High concentration of H_2_ improved mitochondrial biogenesis

**FIGURE 6:**
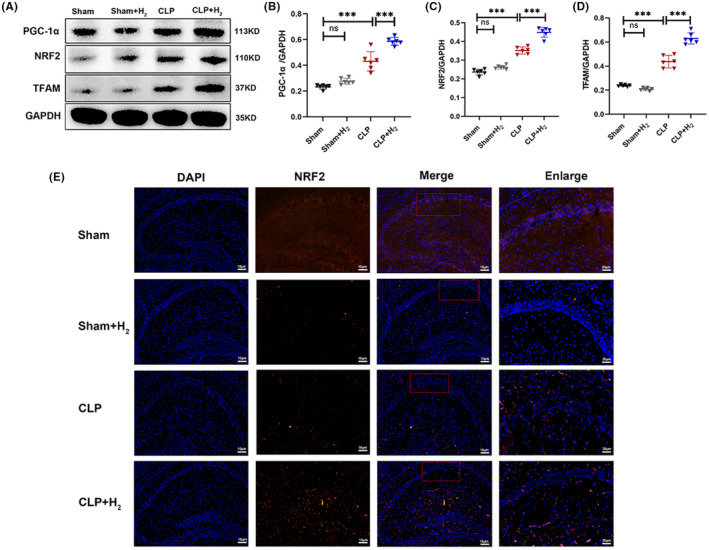
67% H_2_ improved the mitochondrial biogenesis in septic mice. (A–D) The expression of mitochondrial biogenesis‐related proteins (PGC‐1α, NRF2, and TFAM) was assayed by western blotting. (E) NRF2 was observed by immunofluorescence staining. All the data were expressed as means ± SD (*n* = 6 per group). ***p < 0.01 compared with two groups.

Mitochondrial biogenesis regulates the generation of new mitochondria. Relevant proteins in hippocampus tissue were measured 24 h after casting. As shown in Figure 6A–D, the protein expression levels of PGC‐1α, NRF2, and TFAM significantly increased in the CLP group compared to the Sham group (*p* < 0.001). This indicates that septic stimulation induced an active phase and increased biosynthesis of hippocampal tissue mitochondria. Inhalation of 67% H_2_ further up‐regulated the expression levels of these proteins compared to the CLP group (*p* < 0.001). Moreover, NRF2 was labeled with red fluorescence and directly observed by immunofluorescence (Figure 6E). Compared with the Sham group, the expression of NRF2 in the CLP group was significantly increased, while the expression of NRF2 increased further after H_2_ treatment. These results indicated that high concentrations of H_2_ enhanced hippocampal mitochondrial function and alleviated sepsis‐induced brain injury in mice.

## 4 DISCUSSION

In this study, we found that 67% H_2_ could improve the survival rate of septic mice, alleviate the pathological damage of brain tissue as well as cognitive dysfunction, and reduce the inflammatory factors as well as redox imbalances. This beneficial effect was related to the improvement of mitochondria dynamics and biogenesis.

In sepsis, the brain tissue is one of the most vulnerable target organs. Clinically, SAE has a high fatality rate, and survivors often present with chronic autonomic dysfunction, delirium or cognitive impairment of varying degrees.^15^ The mortality rate of CLP model mice established in this study was as high as 50% and the cognitive function of mice was impaired, which was consistent with previous studies.^16^ Inhalation of 67% H_2_ could effectively improve the survival rate and had a protective effect on cognitive function.

Studies have found that cognitive dysfunction in sepsis patients is closely related to hippocampal inflammation and pathological damage.^17^, ^18^ Proinflammatory cytokines play a key role in the pathophysiology of SAE in critically ill patients.^19^, ^20^ Elevated levels of proinflammatory factors lead to neuronal apoptosis, necrosis,^21^ axon damage, brain edema,^22^ inhibition of neurotransmitter transporters, and breakdown of the blood–brain barrier (BBB).^23^ In this study, elevated hippocampal inflammatory factors such as TNF‐α, IL‐1β, and HGMB1 were observed in mice with sepsis, as well as neuropathological changes in the CA1 region of the hippocampus, including disordered neuronal arrangement, reduced structural density, and reduced number of normal neurons. Chen et al. shown that H_2_ alleviates hippocampal damage in a variety of neurological diseases, primarily by regulating inflammatory responses and alleviating histological damage.^17^ This is consistent with our findings that 67% H_2_ treatment improves hippocampal inflammatory cytokine levels and pathological tissue.

Brain tissue has lower antioxidant defense level and higher oxygen consumption compared with other organs. Therefore, it is more susceptible to oxidative damage in sepsis.^24^ The imbalance of oxidative stress can disrupt cellular respiration and lead to abnormal metabolism, and the free radicals produced can induce inflammatory mediators, causing the breakdown of the blood–brain barrier and secondary brain damage.^25^ Experimental and clinical studies have shown that the increased production of ROS in sepsis is associated with the consumption of antioxidants.^24^ CAT and SOD are crucial antioxidant enzymes that reflect the body's antioxidant levels and damage capacity.^26^, ^27^ In our study, SOD and CAT were significantly decreased in the hippocampus of CLP mice, while inhalation of 67% H_2_ significantly improved antioxidant capacity. Furthermore, ROS can impair ATP synthesis by inducing mitochondrial structural changes that lead to loss of mitochondrial enzyme activity, which is associated with acute brain dysfunction in SAE patients.^28^ At the same time, the mitochondrial permeable pore (mPTP) opens abnormally due to the imbalance of cellular oxidative stress. Excessive water and electrolyte enter the mitochondrial matrix, resulting in mitochondrial swelling, mitochondrial outer membrane rupture, MMP depletion, and a large amount of oxygen free radicals are produced.^29^ Excessive ROS causes damage to mitochondrial proteins, and ROS clearance is impaired, which may cause a vicious cycle and further lead to neuronal injury.^29^ Our previous studies^30^, ^31^, ^32^, ^33^ have shown that 2% H_2_ has antioxidant and anti‐inflammatory effects. In addition to protecting multiple organs in sepsis, it can also improve mitochondrial function.^31^ In this study, we found that 67% H_2_ also had the same function, improving MMP and ATP production.

Mitochondria play a vital role in neuronal function. Once changes in the mitochondrial dynamics occur, including fission and fusion, harmful effects may occur.^34^ These processes are regulated by key proteins, such as Drp1, Mfn1, and Mfn2. Drp1 and mitochondrial fission protein 1 are associated with mitochondrial fission, while mitofusin, Mfn1, Mfn2, and optic atrophy protein 1 (OPA1) are involved in mitochondrial fusion.^35^ In the SAE model, Haileselassie et al.^36^ observed that the disruption of the cell respiratory chain under LPS stimulation, leading to the loss of MMP, resulting in an increase in Drp1 and the recruitment of p53 to the mitochondrial outer membrane, followed by the initiation of the cell death pathway. At the same time, Li et al. have shown that HO‐1 can reduce sepsis‐induced renal injury by regulating the expression of Drp1 and up‐regulating the expression of Mfn2.^37^ Consistent with references, Drp1 increased and Mfn2 decreased in the hippocampus of sepsis mice. However, 67% H_2_ treatment can improve the expression of the above proteins, suggesting that 67% H_2_ has an improved effect on mitochondrial dynamics.

Mitochondrial biogenesis, described as the growth and division of mitochondria, improves survival in patients with sepsis by producing new mitochondria, while inhibition of mitochondrial biogenesis worsens prognosis.^38^, ^39^ It is currently widely believed that damaged mitochondrial clearance in sepsis organs can be compensated by increased mitochondrial biogenesis to produce new mitochondria, which may be a key reason for improving survival rates and reversing organ damage in sepsis patients.^40^ Mitochondrial biogenesis is regulated by nuclear and mitochondrial factors such as PGC‐1α, NRF1, NRF2, and TFAM. Specifically, PGC‐1α activates NRF1 and NRF2, which regulate mitochondrial biogenesis by overseeing the expression of nuclear‐encoded proteins involved in mitochondrial respiratory function and translation.^41^ Moreover, PGC‐1α activation triggers TFAM translocation from the cytosol to the mitochondria, where it binds to mtDNA and governs its transcription and replication.^42^ Up‐regulation of PGC‐1α has been shown to reduce the production of and mitochondrial ROS, as well as levels of intracellular inflammatory factors.^43^, ^44^ Zhao et al. observed that the biogenesis of mitochondria in cerebral cortical astrocytes was strengthened to cope with the increased energy demand of astrocytes under sepsis conditions, and finally the ultrastructure of mitochondria was restored under mild injury.^45^ This is consistent with our findings that the expression levels of mitochondrial biosynthetic proteins such as PGC‐1α, NRF2, and TFAM were significantly elevated in mice with sepsis, and the expression of biosynthetic proteins was higher after 67% H_2_ treatment. These results suggest that 67% of H_2_ improves SAE by promoting mitochondrial biogenesis.

In 2007, Ohsawa et al.^46^ first reported that hydrogen (H_2_), as a therapeutic antioxidant, can effectively remove the most cytotoxic oxygen free radicals and has a protective effect on cerebral ischemia–reperfusion injury. Our previous studies have also shown that 2% H_2_ has a protective effect on multiple organs of sepsis, including intestinal injury,^32^ lung injury,^31^ liver injury,^33^ and brain injury.^16^ Although 2% H_2_ has a protective effect on multiple organs in sepsis, this does not mean that 2% H_2_ is the optimal therapeutic concentration, and the optimal concentration is still unknown.

In the past, there was a view that high concentration of hydrogen was easy to explode and its safety was poor. However, with the development of advanced high concentration H_2_ therapeutic devices, 66.7% hydrogen and 33.3% oxygen can be produced by electrolyzing water, and the safety of use is ensured through safety mechanisms such as sealing loop and concentration monitoring feedback. Using these devices, high concentration H_2_ (67%) has been used to treat a variety of diseases, leading to favorable outcomes.^6^, ^14^, ^47^ Inhaling high concentrations of H_2_ boosts its antioxidant effect by theoretically elevating its concentration in tissues, cells, and organelles.^14^ Jun et al.'s study showed that 67% of H_2_ inhalation can be maintained in tissues for a better duration than 4% H_2_ and was more protective against acute kidney injury.^47^ In addition, Xie et al.'s study showed that inhalation of 67% H_2_ could better improve 7‐day survival rate and sepsis induced liver, kidney and lung injury in mice than 2% H_2_, which may be related to the more activation of Nrf2 signaling pathway.^14^ This suggested that the therapeutic effect of high concentration H_2_ inhalation is better than that of low concentration H_2_ inhalation. Therefore, we investigated whether high levels of H_2_ have a protective effect on septic brain injury.

There are limitations in our study. First, we only use a dose of 67% H_2_, so the dose effect and optimal dose are unknown. Second, the current treatment of inhalation of high concentrations of H_2_ is relatively mature, but H_2_ still has the risk of explosion. Finally, we only explored the protective effects of high concentration H_2_ on the brain, liver and kidney, and whether it has protective effects on other organs is worth further exploration.

## 5 CONCLUSION

Inhalation of high concentration (67%) of H_2_ in septic mice improved survival and reduced neuronal injury. Its mechanism might be mediated by enhanced mitochondrial biogenesis and dynamics.

## AUTHOR CONTRIBUTIONS

Conceptualization, Yan Cui, Shuqi Meng, Nannan Zhang, Jingya Liu, Lina Zheng, and Wanjie Ma; data curation, Nannan Zhang, Shuqi Meng, Yu Song, Zhiwei Wang and Jianfeng Liu; formal analysis, Yan Cui; funding acquisition, Yan Cui and Keliang Xie; investigation, Nannan Zhang, Shuqi Meng, Lina Zheng, Wanjie Ma, Yu Song and Zhiwei Wang; methodology, Yan Cui, Nannan Zhang, Shuqi Meng, Lina Zheng and Wanjie Ma; project administration, Keliang Xie; Software, Lina Zheng, Yuehao Shen and Wanjie Ma; supervision, Keliang Xie; Validation, Yu Song, Zhiwei Wang and Jianfeng Liu; writing—original draft, Nannan Zhang and Shuqi Meng; Writing—review and editing, Yan Cui.

## FUNDING INFORMATION

This study was supported by a grant from the research project of Tianjin Municipal Education Commission (2022KJ193 to Yan Cui), Tianjin Medical University General Hospital Clinical Research Program (TJWJ2024XK006 to Keliang Xie), and Tianjin Health Science and Technology Project (ZX‐1200004002‐2024‐4118 to Keliang Xie), Tianjin, China.

## CONFLICT OF INTEREST STATEMENT

The authors declare that they have no known competing financial interest or personal relationships that could have appeared to influence the work reported in this paper.

## INSTITUTIONAL REVIEW BOARD STATEMENT

This study was approved by the Animal Ethics Committee of the Tianjin Medical University General Hospital with approval number (grant no. IRB2022‐DWFL‐587).

## Supporting information


Figures S1‐S2.

## Data Availability

Data will be made available on request.

## References

[1] Angus DC , Bindman AB . Achieving diagnostic excellence for sepsis. JAMA. 2022;327(2):117‐118. 34940801 10.1001/jama.2021.23916

[2] Sonneville R , Benghanem S , Jeantin L , et al. The spectrum of sepsis‐associated encephalopathy: a clinical perspective. Crit Care. 2023;27(1):386. 37798769 10.1186/s13054-023-04655-8 PMC10552444

[3] Chen AX , Simpson SQ , Pallin DJ . Sepsis Guidelines. N Engl J Med. 2019;380(14):1369‐1371. 30943343 10.1056/NEJMclde1815472

[4] Patel BK , Wolfe KS , Patel SB , et al. Effect of early mobilisation on long‐term cognitive impairment in critical illness in the USA: a randomised controlled trial. Lancet Respir Med. 2023;11(6):563‐572. 36693400 10.1016/S2213-2600(22)00489-1 PMC10238598

[5] Borcherding N , Brestoff JR . The power and potential of mitochondria transfer. Nature. 2023;623(7986):283‐291. 37938702 10.1038/s41586-023-06537-z PMC11590279

[6] Zhao N , Sun R , Cui Y , et al. High concentration hydrogen mitigates sepsis‐induced acute lung injury in mice by alleviating mitochondrial fission and dysfunction. J Pers Med. 2023;13(2):244. 36836478 10.3390/jpm13020244 PMC9966938

[7] Wang Y , McLean AS . The role of mitochondria in the immune response in critical illness. Crit Care. 2022;26(1):80. 35337333 10.1186/s13054-022-03908-2 PMC8957137

[8] Chen H , Lin H , Dong B , Wang Y , Yu Y , Xie K . Hydrogen alleviates cell damage and acute lung injury in sepsis via PINK1/Parkin‐mediated mitophagy. Inflamm Res. 2021;70(8):915‐930. 34244821 10.1007/s00011-021-01481-y

[9] Chan DC . Mitochondrial dynamics and its involvement in disease. Annu Rev Pathol. 2020;15:235‐259. 31585519 10.1146/annurev-pathmechdis-012419-032711

[10] Anitha A , Thanseem I , Iype M , Thomas SV . Mitochondrial dysfunction in cognitive neurodevelopmental disorders: cause or effect? Mitochondrion. 2023;69:18‐32. 36621534 10.1016/j.mito.2023.01.002

[11] Zhang Y , Chen J , Wu H , et al. Hydrogen regulates mitochondrial quality to protect glial cells and alleviates sepsis‐associated encephalopathy by Nrf2/YY1 complex promoting HO‐1 expression. Int Immunopharmacol. 2023;118:110009. 36963264 10.1016/j.intimp.2023.110009

[12] Johnsen HM , Hiorth M , Klaveness J . Molecular hydrogen therapy‐a review on clinical studies and outcomes. Molecules. 2023;28(23):7785. 38067515 10.3390/molecules28237785 PMC10707987

[13] Qi B , Yu Y , Wang Y , Wang Y , Yu Y , Xie K . Perspective of molecular hydrogen in the treatment of sepsis. Curr Pharm Des. 2021;27(5):667‐678. 32912119 10.2174/1381612826666200909124936

[14] Sun R , Zhao N , Wang Y , et al. High concentration of hydrogen gas alleviates lipopolysaccharide‐induced lung injury via activating Nrf2 signaling pathway in mice. Int Immunopharmacol. 2021;101(Pt B):108198. 34634688 10.1016/j.intimp.2021.108198

[15] Ji MH , Gao YZ , Shi CN , Wu XM , Yang JJ . Acute and long‐term cognitive impairment following sepsis: mechanism and prevention. Expert Rev Neurother. 2023;23(10):931‐943. 37615511 10.1080/14737175.2023.2250917

[16] Qi B , Song Y , Chen C , et al. Molecular hydrogen attenuates sepsis‐induced cognitive dysfunction through regulation of tau phosphorylation. Int Immunopharmacol. 2023;114:109603. 36538853 10.1016/j.intimp.2022.109603

[17] Chen H , Dong B , Shi Y , Yu Y , Xie K . Hydrogen alleviates neuronal injury and neuroinflammation induced by microglial activation via the nuclear factor erythroid 2‐related factor 2 pathway in sepsis‐associated encephalopathy. Neuroscience. 2021;466:87‐100. 33992722 10.1016/j.neuroscience.2021.05.003

[18] Jiang J , Zou Y , Xie C , et al. Oxytocin alleviates cognitive and memory impairments by decreasing hippocampal microglial activation and synaptic defects via OXTR/ERK/STAT3 pathway in a mouse model of sepsis‐associated encephalopathy. Brain Behav Immun. 2023;114:195‐213. 37648002 10.1016/j.bbi.2023.08.023

[19] Tauber SC , Djukic M , Gossner J , Eiffert H , Brück W , Nau R . Sepsis‐associated encephalopathy and septic encephalitis: an update. Expert Rev Anti‐Infect Ther. 2021;19(2):215‐231. 32808580 10.1080/14787210.2020.1812384

[20] Mazeraud A , Righy C , Bouchereau E , Benghanem S , Bozza FA , Sharshar T . Septic‐associated encephalopathy: a comprehensive review. Neurotherapeutics. 2020;17(2):392‐403. 32378026 10.1007/s13311-020-00862-1 PMC7283452

[21] Kneussel M , Friese MA . SnapShot: neuronal dysfunction in inflammation. Neuron. 2021;109(10):1754‐1754.e1. 34015268 10.1016/j.neuron.2021.03.005

[22] Wang P , Yan J , Shi Q , et al. Relationship between nonhepatic serum ammonia levels and sepsis‐associated encephalopathy: a retrospective cohort study. Emerg Med Int. 2023;2023:6676033. 37869361 10.1155/2023/6676033 PMC10590267

[23] Galea I . The blood‐brain barrier in systemic infection and inflammation. Cell Mol Immunol. 2021;18(11):2489‐2501. 34594000 10.1038/s41423-021-00757-x PMC8481764

[24] Ren C , Yao RQ , Zhang H , Feng YW , Yao YM . Sepsis‐associated encephalopathy: a vicious cycle of immunosuppression. J Neuroinflammation. 2020;17(1):14. 31924221 10.1186/s12974-020-1701-3 PMC6953314

[25] Catarina AV , Branchini G , Bettoni L , De Oliveira JR , Nunes FB . Sepsis‐associated encephalopathy: from pathophysiology to progress in experimental studies. Mol Neurobiol. 2021;58(6):2770‐2779. 33495934 10.1007/s12035-021-02303-2

[26] Aleksandrova K , Koelman L , Rodrigues CE . Dietary patterns and biomarkers of oxidative stress and inflammation: a systematic review of observational and intervention studies. Redox Biol. 2021;42:101869. 33541846 10.1016/j.redox.2021.101869 PMC8113044

[27] Wang Y , Lv W , Li Y , Liu D , He X , Liu T . Ampelopsin improves cognitive impairment in Alzheimer's disease and effects of inflammatory cytokines and oxidative stress in the hippocampus. Curr Alzheimer Res. 2020;17(1):44‐51. 31797758 10.2174/1567205016666191203153447

[28] Berg RM , Møller K , Bailey DM . Neuro‐oxidative‐nitrosative stress in sepsis. J Cereb Blood Flow Metab. 2011;31(7):1532‐1544. 21487413 10.1038/jcbfm.2011.48 PMC3137474

[29] Quoilin C , Mouithys‐Mickalad A , Lécart S , Fontaine‐Aupart MP , Hoebeke M . Evidence of oxidative stress and mitochondrial respiratory chain dysfunction in an in vitro model of sepsis‐induced kidney injury. Biochim Biophys Acta. 2014;1837(10):1790‐1800. 25019585 10.1016/j.bbabio.2014.07.005

[30] Cui Y , Li Y , Meng S , Song Y , Xie K . Molecular hydrogen attenuates sepsis‐induced cardiomyopathy in mice by promoting autophagy. BMC Anesthesiol. 2024;24(1):72. 38395800 10.1186/s12871-024-02462-4 PMC10885652

[31] Xie K , Wang Y , Yin L , et al. Hydrogen gas alleviates sepsis‐induced brain injury by improving mitochondrial biogenesis through the activation of PGC‐α in mice. Shock. 2021;55(1):100‐109. 32590694 10.1097/SHK.0000000000001594

[32] Chen HG , Han HZ , Li Y , Yu YH , Xie KL . Hydrogen alleviated organ injury and dysfunction in sepsis: the role of cross‐talk between autophagy and endoplasmic reticulum stress: experimental research. Int Immunopharmacol. 2020;78:106049. 31830624 10.1016/j.intimp.2019.106049

[33] Yan M , Yu Y , Mao X , et al. Hydrogen gas inhalation attenuates sepsis‐induced liver injury in a FUNDC1‐dependent manner. Int Immunopharmacol. 2019;71:61‐67. 30877875 10.1016/j.intimp.2019.03.021

[34] Dumbuya JS , Li S , Liang L , Zeng Q . Paediatric sepsis‐associated encephalopathy (SAE): a comprehensive review. Mol Med. 2023;29(1):27 Published 2023 Feb 23. 36823611 10.1186/s10020-023-00621-w PMC9951490

[35] Song J , Herrmann JM , Becker T . Quality control of the mitochondrial proteome. Nat Rev Mol Cell Biol. 2021;22(1):54‐70. 33093673 10.1038/s41580-020-00300-2

[36] Haileselassie B , Joshi AU , Minhas PS , Mukherjee R , Andreasson KI , Mochly‐Rosen D . Mitochondrial dysfunction mediated through dynamin‐related protein 1 (Drp1) propagates impairment in blood brain barrier in septic encephalopathy. J Neuroinflammation. 2020;17(1):36. 31987040 10.1186/s12974-019-1689-8 PMC6986002

[37] Shi J , Yu J , Zhang Y , et al. PI3K/Akt pathway‐mediated HO‐1 induction regulates mitochondrial quality control and attenuates endotoxin‐induced acute lung injury. Lab Investig. 2019;99(12):1795‐1809. 31570770 10.1038/s41374-019-0286-x

[38] Popov LD . Mitochondrial biogenesis: an update. J Cell Mol Med. 2020;24(9):4892‐4899. 32279443 10.1111/jcmm.15194 PMC7205802

[39] Liu L , Li Y , Chen G , Chen Q . Crosstalk between mitochondrial biogenesis and mitophagy to maintain mitochondrial homeostasis. J Biomed Sci. 2023;30(1):86. 37821940 10.1186/s12929-023-00975-7 PMC10568841

[40] Lira Chavez FM , Gartzke LP , van Beuningen FE , et al. Restoring the infected powerhouse: mitochondrial quality control in sepsis. Redox Biol. 2023;68:102968. 38039825 10.1016/j.redox.2023.102968 PMC10711241

[41] Supinski GS , Schroder EA , Callahan LA . Mitochondria and critical illness. Chest. 2020;157(2):310‐322. 31494084 10.1016/j.chest.2019.08.2182 PMC7005375

[42] Kummer E , Ban N . Mechanisms and regulation of protein synthesis in mitochondria. Nat Rev Mol Cell Biol. 2021;22(5):307‐325. 33594280 10.1038/s41580-021-00332-2

[43] McCreath G , Scullion MM , Lowes DA , Webster NR , Galley HF . Pharmacological activation of endogenous protective pathways against oxidative stress under conditions of sepsis. Br J Anaesth. 2016;116(1):131‐139. 26675956 10.1093/bja/aev400

[44] Abu Shelbayeh O , Arroum T , Morris S , Busch KB . PGC‐1α is a master regulator of mitochondrial lifecycle and ROS stress response. Antioxidants (Basel). 2023;12(5):1075. 37237941 10.3390/antiox12051075 PMC10215733

[45] Zhao YZ , Gao ZY , Ma LQ , Zhuang YY , Guan FL . Research on biogenesis of mitochondria in astrocytes in sepsis‐associated encephalopathy models. Eur Rev Med Pharmacol Sci. 2017;21(17):3924‐3934. 28975969

[46] Kurokawa R , Hirano SI , Ichikawa Y , Matsuo G , Takefuji Y . Preventing explosions of hydrogen gas inhalers. Med Gas Res. 2019;9(3):160‐162. 31552881 10.4103/2045-9912.266996 PMC6779006

[47] Xue JL , Liu BY , Zhao M , et al. Inhalation of 4% and 67% hydrogen ameliorates oxidative stress, inflammation, apoptosis, and necroptosis in a rat model of glycerol‐induced acute kidney injury. Med Gas Res. 2023;13(2):78‐88. 36204787 10.4103/2045-9912.345169 PMC9555022

