# Supplementary material for: High‐concentration hydrogen inhalation mitigates sepsis‐associated encephalopathy in mice by improving mitochondrial dynamics
Source: CNS Neurosci Ther. 2024 Sep 11;30(9):e70021. doi: 10.1111/cns.70021 (PMC11388582; doi:10.1111/cns.70021)
Supplement: Supplementary file 1 — Figures S1‐S2. [file CNS-30-e70021-s001.pdf]

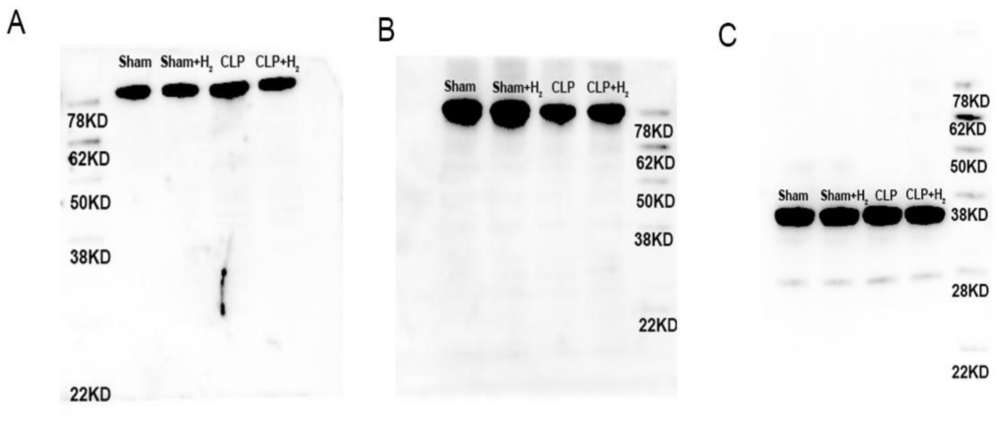

Supplementary Figure 1. The whole membrane protein in Figure 5. (A) Drp1 protein. (B) MFN2 protein. (C) GAPDH protein.

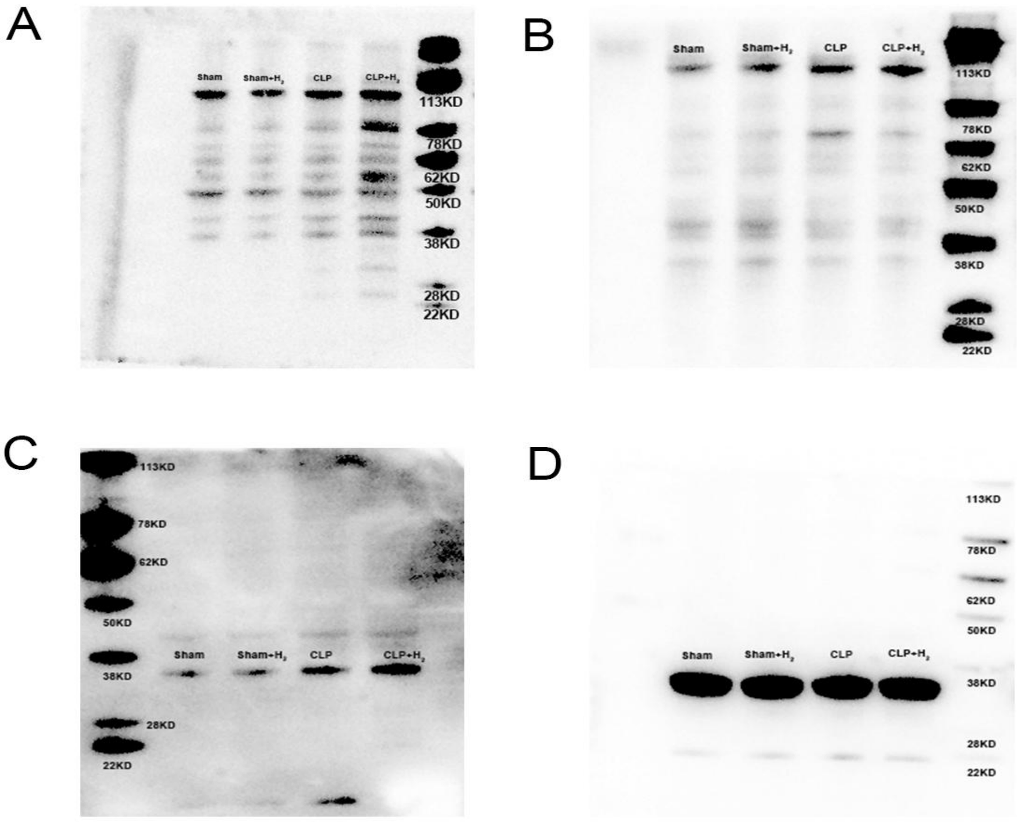

Supplementary Figure 2. The whole membrane protein in Figure 6. (A) PGC-1α protein. (B) NRF2 protein. (C) TFAM protein. (D) GAPDH protein.
